# Optimizing Oxygen Delivery by Low-Flow Nasal Cannula to Small Infants: A Bench Study

**DOI:** 10.3390/diagnostics14090889

**Published:** 2024-04-24

**Authors:** Aris Bertzouanis, Xenophon Sinopidis, Polyxeni Pelekouda, Ageliki Karatza, Gabriel Dimitriou, Sotirios Fouzas

**Affiliations:** Department of Pediatrics, Medical School, University of Patras, 26500 Patras, Greece; xsinopid@upatras.gr (X.S.); ppelekouda@upatras.gr (P.P.); karatza@upatras.gr (A.K.); gdim@upatras.gr (G.D.); sfouzas@upatras.gr (S.F.)

**Keywords:** low-flow nasal cannula, oxygen, infants

## Abstract

Background: In infants treated with a low-flow nasal cannula (LFNC), the oxygen concentration delivered to the lungs (i.e., the effective FiO_2_) is difficult to estimate. The existing mathematical formulas rely on important assumptions regarding the values of respiratory parameters and, thus, may be inaccurate. We aimed to assess oxygen delivery by LFNC to small infants using realistic simulations on a mechanical breathing model. Methods: A mechanical breathing simulator (infant upper-airway replica, single-space breathing compartment, electric motor, microcontroller) was developed. Breathing simulations (*n* = 1200) were performed at various tidal volume (VT), inspiratory time (Ti), and respiratory rate (RR) combinations and different cannula flows. Results: Minute ventilation (MV) was the most significant predictor of effective FiO_2_. FiO_2_ was higher at lower VT and higher Ti values. Benaron and Benitz’s formula underestimated the effective FiO_2_ at lower MV values, while Finer’s formula significantly overestimated it. A set of predictive FiO_2_ charts was developed based on cannula flow, infant body weight, and RR. Conclusions: The effective FiO_2_ delivered by LFNC to small infants critically depends on VT, Ti, and RR. However, since VT and Ti values are not available in clinical practice, the existing mathematical formulas may be inaccurate. Our novel predictive FiO_2_ charts could assist in optimizing oxygen delivery by LFNC using easy-to-obtain parameters, such as infant body weight and RR.

## 1. Introduction

Oxygen administration to small infants via low-flow nasal cannula (LFNC; gas flow ≤ 2 L/min) is a standard practice in neonatal and pediatric care [1]. However, the fraction of inspired oxygen (FiO_2_) delivered to the lungs, known as the effective FiO_2_, is difficult to estimate because it depends on many factors: the oxygen concentration in the supplied gas, the cannula flow, and the dynamics of respiration (tidal volume—VT, inspiratory time—Ti, expiratory time—Te) [2,3,4]. Indeed, it has been shown that the hypopharyngeal FiO_2_—a surrogate of the effective FiO_2_—may be extremely variable in small infants receiving oxygen via LFNC [5], even at very low cannula flows (e.g., FiO_2_ 23–54% at 0.1 L/min 100% oxygen flow) [6]. In preterm newborns, uncontrolled oxygen supply may lead to hyperoxemia, which has been associated with retinopathy of prematurity (ROP) and bronchopulmonary dysplasia, while exposure to high FiO_2_ levels may result in atelectasis, interstitial edema, and ventilation/perfusion mismatch, irrespective of the infant’s age and maturity [7,8]. Therefore, optimizing the oxygen delivered by LFNC in clinical practice is important [9].

**Figure 1:**
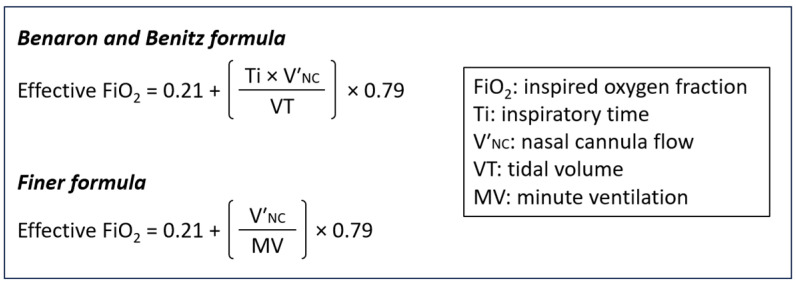
Existing mathematical formulas to calculate the effective FiO_2_ in infants receiving oxygen through a low-flow nasal cannula. Both equations are adapted for a 100% oxygen supply.

In this regard, Benaron and Benitz [3] devised a mathematical model to calculate the effective FiO_2_ based on cannula flow and infant VT and Ti (Figure 1). Similarly, Finner et al. [4] introduced a mathematical formula that is based on cannula flow and infant minute ventilation (MV) (Figure 1). Although the FiO_2_ estimated by these formulas has been shown to correlate well with hypopharyngeal FiO_2_ measurements [3,4], their accuracy critically depends on VT, Ti, and MV values [3,4], which cannot be routinely measured in clinical practice. In the STOP-ROP study [10], a randomized, controlled trial that explored the relationship between oxygen supplementation and ROP in preterm infants, Benaron and Benitz’s formula was used to calculate the effective FiO_2_ assuming a fixed Ti of 300 ms and a fixed VT of 5 mL/kg [11]. The conversion tables of the STOP-ROP study [11], as well as Finner’s formula assuming a fixed VT of 5.5 mL/kg [4], are widely used in clinical practice currently [9]. Nevertheless, a recent study showed that the effective FiO_2_ is difficult to estimate using simple mathematical equations with arbitrarily fixed respiratory parameters [12].

The aim of the present study was to assess oxygen delivery by LFNC to small infants using an upper-airway replica and realistic breathing simulations through a mechanical lung model. We hypothesized that for the same cannula flow, the effective FiO_2_ would be significantly influenced by changes in respiratory parameters, thus resulting in variable oxygen delivery to the lungs. We also aimed to incorporate the FiO_2_ variability in simplified predictive charts that could assist healthcare professionals in optimizing oxygen delivery by LFNC to small infants. 

## 2. Materials and Methods

### 2.1. Experimental Setup

**Figure 2:**
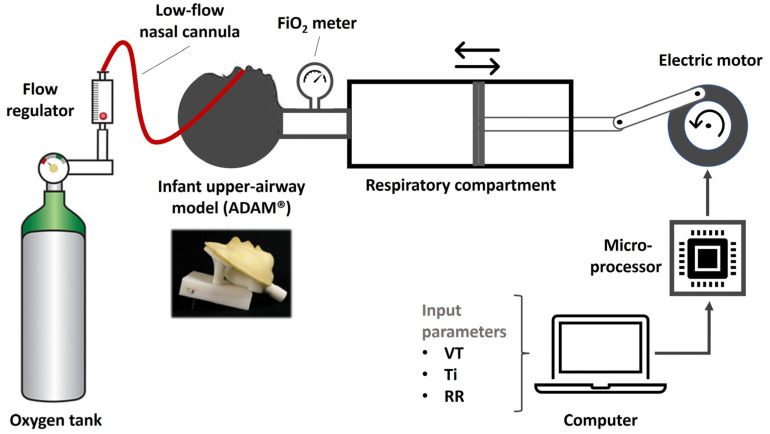
Experimental setup. VT: tidal volume, Ti: inspiratory time, RR: respiratory rate.

A mechanical infant breathing simulator (Figure 2) was developed as described in the GitHub repository: https://github.com/arisberd/Infant-breath-mechanical-simulator (accessed on 2 March 2024). Briefly, the lung simulator consisted of a single-space breathing compartment (100 mL Hans Rudolph calibration syringe, model 5510, Hans Rudolph Inc., Kansas City, MO, USA) connected to an ADAM infant upper-airway replica [13]. The replica was slightly modified by cutting the airway at the glottis level to reduce excess (subglottic) dead space. The volume of the replica’s air passages was subsequently measured by a water displacement method (10 consecutive measurements) and found to be 5.2 ± 0.1 mL. A pliant plastic rod (diameter 5 mm, length 50 mm, volume 1 mL) was introduced to the replica’s air passages to reduce dead space when the simulated VT was <10 mL. Conversely, an expandable tube was placed between the ADAM model and the breathing compartment to increase the dead space volume of the upper airways accordingly [14] when the simulated VT was >50 mL. The syringe’s piston was connected to a stepper motor (Jiangsu Wantai Motor Co., Changzhou, China) controlled using an Arduino Uno R3 board [15]. Breathing simulations, based on various VT, Ti, and RR combinations (see below), were set in a personal computer and uploaded to the Arduino microcontroller. The FiO_2_ was measured at the tip of the syringe using an AΧ300 oxygen analyzer (Teledyne, City of Industry, CA, USA). The analyzer was calibrated at the beginning of each cycle of simulations and every two hours of operation. A commercially available neonatal/infant-sized LFNC was mounted on the ADAM model according to our setting guidelines. The LFNC was connected to a Debson TM2 flowmeter (Technologie Medicale, Noisy Le Sec, France; flow range 0.1–1 L/min, 0.1 L/min resolution) supplied with 100% oxygen from an oxygen tank. 

### 2.2. Simulations

All simulations were performed at an ambient temperature of 21–24 °C and relative humidity of 50–65%. Cannula flows and respiratory parameters were validated before each experiment using the SmartLab Data Acquisition System with Insight Software version 3.2.0 (Hans Rudolph Inc.). 

Breathing simulation scenarios were based on previously published infant tidal-breathing measurements [16,17,18,19]. The range of tested values for each respiratory parameter is shown in Table 1. Each scenario consisted of a fixed combination of VT, respiratory rate (RR), and Ti (input variables), which produced a respiratory cycle with unique characteristics (output variables). The VT was simulated at increments of 5 mL for volumes between 5 and 20 mL, followed by increments of 10 mL up to the volume of 80 mL. The RR was simulated at intervals of 10 breaths/min and the Ti at intervals of 200 ms. The input variables (i.e., VT, RR, and Ti) were shuffled to obtain all possible combinations within the set ranges (Table 1), resulting in Ti/Te ratios between 0.5 and 1.5 and MV values between 150 and 6400 mL/min. Four nasal cannula flows were tested: 0.1, 0.3, 0.5, and 1 L/min, all at 100% oxygen supply. The experiment was continued until the measured FiO_2_ (Figure 2) was stabilized. Each simulation scenario was repeated in triplicate, resulting in a total of 1200 experiments. Between the scenarios, the nasal cannula was disconnected from the oxygen supply to allow for the oxygen wash-out of the mechanical simulator (i.e., until the measured FiO_2_ returned to 21%). 

### 2.3. Statistics and Charts

The average values (three experiments) of effective FiO_2_ were recorded and plotted against VT and Ti for different cannula flows. The relationship between effective FiO_2_ and MV was also explored and described by non-linear fitting methods. The difference between the FiO_2_ predicted by Benaron and Benitz’s or Finer’s formula and the FiO_2_ of the experiments was calculated and plotted in relation to MV; the level of statistical significance of these differences was assessed by the Mann–Whitney U test. Effective FiO_2_ plots in relation to RR and infant body weight were constructed, setting the VT at 5 and 7.5 mL/kg. All charts were designed using Microsoft Excel (version 365).

## 3. Results

### 3.1. Determinants of Effective FiO_2_

**Figure 3:**
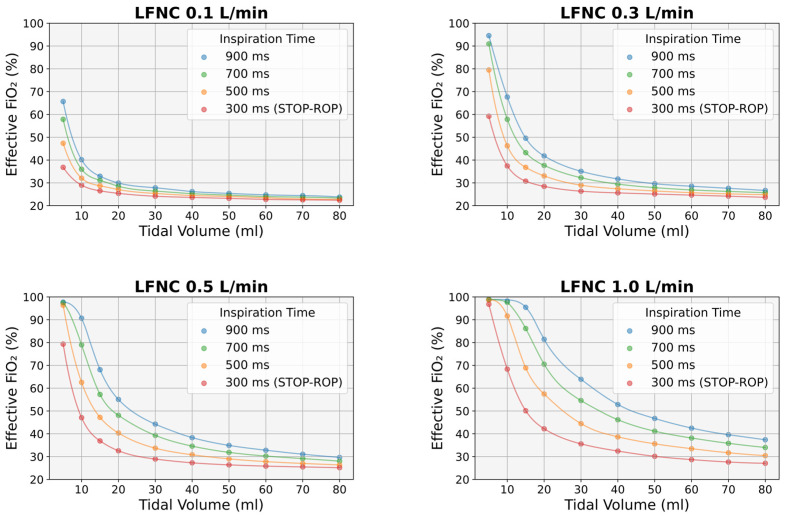
Effective FiO_2_ in relation to tidal volume and inspiratory time at different nasal cannula flows. Each point represents the average FiO_2_ value of six to nine experiments, depending on expiratory time values. Note that an inspiratory time of 300 ms (i.e., the STOP-ROP value [11]) resulted in the lowest FiO_2_ values. LFNC: low-flow nasal cannula.

There was a strong relationship between effective FiO_2_ and VT for different Ti values and at different nasal cannula flows (Figure 3). Effective FiO_2_ was higher at lower VT and higher Ti values, regardless of cannula flow. A Ti of 300 ms resulted in the lowest effective FiO_2_, irrespective of VT and cannula flow (Figure 3). 

More precisely, at a cannula flow of 0.1 L/min and for a VT of 5 mL, the FiO_2_ was 65.7 ± 0.2% for a Ti of 900 ms, 57.8 ± 0.2% for a Ti of 700 ms, 47.4 ± 0.2% for a Ti of 500 ms, and 36.8 ± 0.1% for a Ti of 300 ms. At the same cannula flow and for a VT of 10 mL, the FiO_2_ was 40.1 ± 0.2% for a Ti of 900 ms, 36 ± 0.1% for a Ti of 700 ms, 32 ± 0.1% for a Ti of 500 ms, and 29 ± 0.1% for a Ti of 300 ms, while, for a VT of 40 mL, the FiO_2_ values were 26.2 ± 0.1%, 25.3 ± 0.1%, 24.5 ± 0.1%, and 23.6 ± 0.1%, respectively. For a VT of 50 mL, the effective FiO_2_ ranged between 23.2% and 25.4%, for a VT of 60 mL, between 22.7% and 24.9%, for a VT of 70 mL, between 22.5% and 24.5%, and for a VT of 80 mL, between 22.4% and 23.8% (Figure 3). On the other hand, at a cannula flow of 1.0 L/min and for a VT of 5 mL, the FiO_2_ was 98.9 ± 0.2% for a Ti of 900 ms, 97.8 ± 0.2% for a Ti of 700 ms, 98.6 ± 0.2% for a Ti of 500 ms, and 96.8 ± 0.2% for a Ti of 300 ms. At the same cannula flow and for a VT of 10 mL, the FiO_2_ was 98.2 ± 0.2% for a Ti of 900 ms, 97.8 ± 0.2% for a Ti of 700 ms, 91.7 ± 0.2% for a Ti of 500 ms, and 68.4 ± 0.2% for a Ti of 300 ms, while, for a VT of 80 mL, the FiO_2_ values were 37.4 ± 0.1%, 34 ± 0.1%, 30.4 ± 0.1%, and 27.1 ± 0.1%, respectively (Figure 3). 

**Figure 4:**
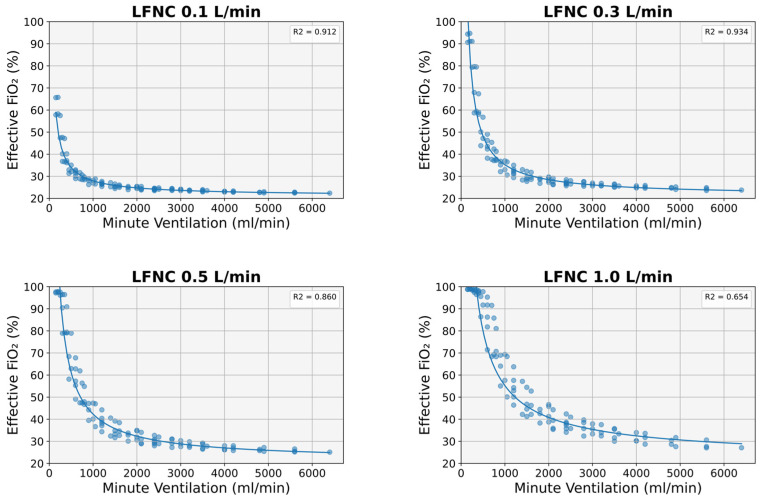
Effective FiO_2_ in relation to minute ventilation at different nasal cannula flows. LFNC: low-flow nasal cannula.

Minute ventilation emerged as the most significant predictor of effective FiO_2_ in all experiments; a power function (FiO_2_(MV) = 21 + k·MV^−a^) could most accurately describe the relationship between FiO_2_ and MV (Figure 4). This relationship was stronger at lower cannula flows (R^2^ 0.9 at 0.1 and 0.3 L/min) but deteriorated significantly at higher flow values (R^2^ 0.860 at 0.5 L/min and 0.654 at 1 L/min) (Figure 4).

### 3.2. Comparison with Existing Mathematical Formulas

**Figure 5:**
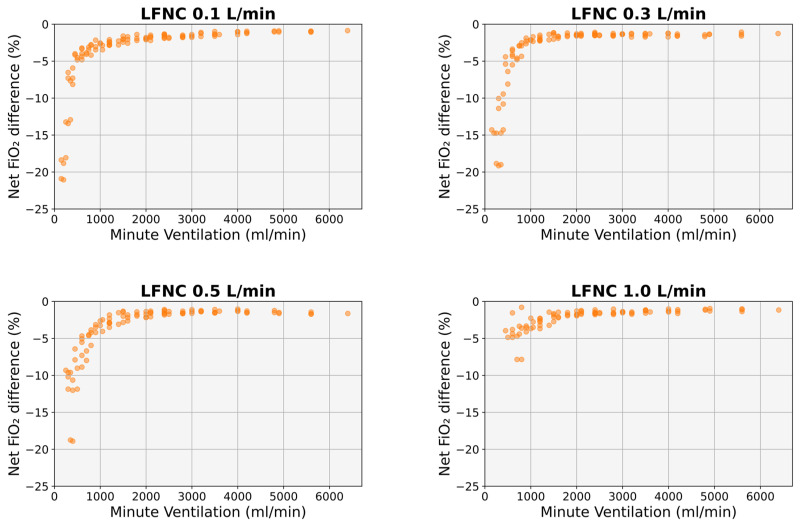
Difference between the FiO_2_ calculated using Benaron and Benitz’s formula [3] and the simulated FiO_2_ for the same MV. LFNC: low-flow nasal cannula, MV: minute ventilation.

Benaron and Benitz’s formula constantly underestimated the effective FiO_2_, particularly for lower MVs and lower cannula flows (Figure 5). For an MV of 150 mL/min, the underestimation was on average 19.6% at a cannula flow of 0.1 L/min (*p* < 0.001), 14.3% at a cannula flow of 0.3 L/min (*p* < 0.01), 4.6% at a cannula flow of 0.5 L/min (*p* > 0.05), and 3.2% at a cannula flow of 1 L/min (*p* > 0.05). The underestimation was less than 5% for MVs > 400 mL/min at a cannula flow of 0.1 L/min, >600 mL/min at a cannula flow of 0.3 L/min, and >800 mL/min at cannula flows of 0.5 and 1 L/min (*p* > 0.05 in all instances). The underestimation was less than 3% for MVs > 1000 mL/min at cannula flows of 0.1 and 0.3 L/min and >1400 mL/min at cannula flows of 0.5 and 1 L/min (*p* > 0.05 in all instances) (Figure 5). 

**Figure 6:**
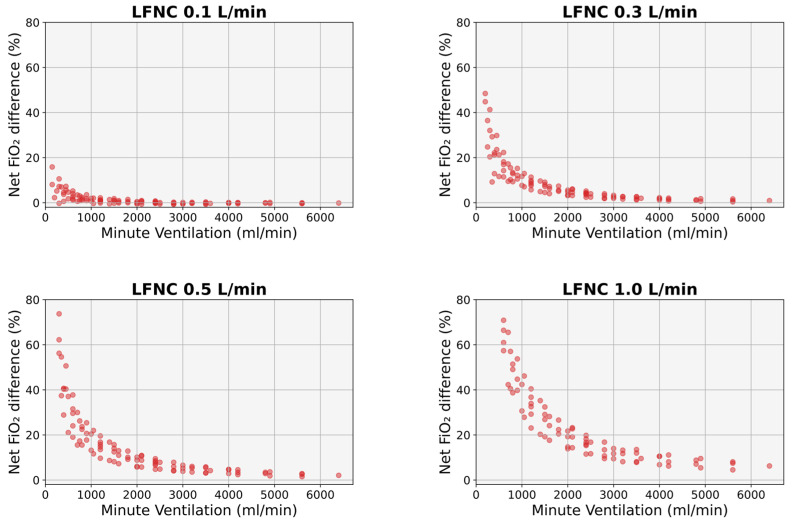
Difference between the FiO_2_ calculated using Finer’s formula [4] and the simulated FiO_2_ for the same MV. LFNC: low-flow nasal cannula, MV: minute ventilation.

Finer’s formula overestimated the simulated FiO_2_, particularly for lower MVs and higher cannula flows (Figure 6). For an MV of 150 mL/min, the overestimation was on average 12% at a cannula flow of 0.1 L/min (*p* < 0.01); at higher cannula flows, the degree of overestimation was falsely higher because Finner’s formula resulted in FiO_2_ values >100% (a known flaw of the respective formula [4]). For an MV of 400 mL/min, the overestimation was on average 2.1% at a cannula flow of 0.1 L/min (*p* > 0.05), 18.8% at a cannula flow of 0.3 L/min (*p* < 0.01), and 36.6% at a cannula flow of 0.5 L/min (*p* < 0.001); at a cannula flow of 1 L/min, Finer’s FiO_2_ values were again >100%, thus resulting in artificially higher differences. The overestimation was less than 5% for MVs > 600 mL/min at a cannula flow of 0.1 L/min, >200 mL/min at a cannula flow of 0.3 L/min, and >3500 mL/min at a cannula flow of 0.5 L/min (*p* > 0.05 in all instances). The overestimation was less than 3% for MVs > 900 mL/min at a cannula flow of 0.1 L/min, >3000 mL/min at a cannula flow of 0.3 L/min, and >5000 mL/min at a cannula flow of 0.5 L/min (*p* > 0.05 in all instances). At a cannula flow of 1 L/min, the overestimation was >5%, irrespective of MV values (*p* < 0.05 for MV values up to 3000 mL/min) (Figure 6).

### 3.3. Predictive FiO_2_ Charts

**Figure 7:**
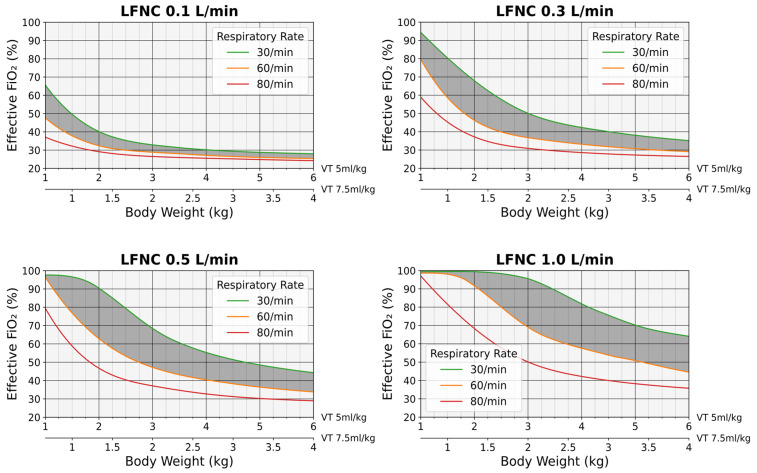
Predictive FiO_2_ charts (100% oxygen supply). LFNC: low-flow nasal cannula, VT: tidal volume. The gray regions depict the values between 30 and 60 breaths per minute.

Based on the simulation results, the FiO_2_ charts shown in Figure 6 were developed. The charts offer an estimate of the effective FiO_2_ in relation to RR and infant body weight, considering a VT of 5 or 7.5 mL/kg (Figure 7). Since the existing mathematical formulas failed to accurately predict the effective FiO_2_ at lower MVs (Figure 5 and Figure 6), the predictive charts were designed for VTs of up to 30 mL, corresponding to MVs of up to 2400 mL/min (Figure 7).

For example, for an infant of 2 kg body weight at a cannula flow of 0.1 L/min, (a) when the VT was estimated at 5 mL/kg and the RR ranged between 30 and 60 breaths/min, the effective FiO_2_ could be between 40.1% and 32.1%, while, for an extreme RR of 80 breaths/min, the effective FiO_2_ could decrease to 29.1%, and (b) when the VT was set to 7.5 mL/kg and the RR ranged between 30 and 60 breaths/min, the effective FiO_2_ could be between 32.9% and 28.5%, whereas, for an RR of 80 breaths/min, the effective FiO_2_ could be as low as 26.5%. Conversely, for the same infant at a cannula flow of 0.5 L/min, (a) when the VT was estimated at 5 mL/kg and the RR ranged between 30 and 60 breaths/min, the effective FiO_2_ could be between 90.9% and 62.8%, while, for an extreme RR of 80 breaths/min, the effective FiO_2_ could decrease to 46.7%, and (b) when the VT was set to 7.5 mL/kg and the RR ranged between 30 and 60 breaths/min, the effective FiO_2_ could be between 68.4% and 47.2%, whereas, for an extreme RR of 80 breaths/min, the effective FiO_2_ could decrease to 36.6% (Figure 7). 

## 4. Discussion

In this study, using an infant upper-airway replica and a custom-built mechanical lung model, we performed realistic simulations to assess the influence of respiration dynamics on the amount of oxygen delivered by LFNC to small infants. We found that the effective FiO_2_ critically depends on respiratory parameters such as VT, Ti, and RR, irrespective of nasal cannula flow. We also showed that the existing predictive formulas cannot accurately estimate effective FiO_2_, especially for infants who attain lower MVs. Finally, we developed practical FiO_2_ charts that may assist healthcare professionals in optimizing oxygen delivery by LFNC using readily available parameters, such as infant body weight and RR. 

Previous relevant work has mainly relied on mathematical modeling [3,4]. Benaron and Benitz presented a formula based on cannula flow and infant VT and Ti (Figure 1). Their main assumption was that the upper airway (i.e., the space consisting of the nasal cavity, nasopharynx, and oropharynx) is negligible and, thus, does not act as an oxygen reservoir [3]. Since VT and Ti cannot be routinely measured, Benaron and Benitz’s formula is used in clinical practice assuming a fixed Ti of 300 ms and a fixed VT of 5 mL/kg [10,11]. In another study, Finer et al. [4] developed an equation to predict hypopharyngeal FiO_2_ measurements based on cannula flow and infant MV (Figure 1). Although their approach lacks Benaron and Benitz’s assumptions, the clinical application of the equation also presumes a fixed VT of 5.5 mL/kg [4]. Finally, in a recent study based on mechanical simulations, Sabz et al. [12] presented a predictive equation that takes into account infant inspiratory flow, a parameter equally challenging to measure in daily practice.

When oxygen is administered via LFNC, the FiO_2_ delivered to the lungs depends on the supplied oxygen concentration, cannula flow, and pattern of respiration [2,3,4]. The latter is expressed by the respiratory parameters VT, Ti, and Te, the combination of which further determines the RR (RR = 60/[Ti + Te]) and MV [MV = VT × RR]. Of note, VT, Ti, and Te are not independent of each other; typically, a higher VT is associated with a prolonged Ti [20], while the Ti/Te ratio cannot exceed a certain range [18]. Moreover, similar values of RR and MV may result from different combinations of Ti, Te, and VT, thus leading to significant FiO_2_ variability. Indeed, a wide range of effective FiO_2_ values for the same RR or MV was noted by us (Figure 3) and others [6,12]. Therefore, since the exact values of VT, Ti, and Te are not available in clinical practice, the accuracy of the FiO_2_ estimated by simple mathematical formulas should be questioned. 

In our study, Benaron and Benitz’s formula underestimated effective FiO_2,_ especially at lower MVs (Figure 5). This is most likely the result of the reservoir effect of the upper airways [20]: when an infant’s end-expiratory flow is lower than the cannula flow, the supplied gas is stored in the upper airways (nasal passages and pharynx), and the available amount of oxygen for the following inspiration is higher than anticipated [20]. The lower the end-expiratory flow and the higher the relative volume of the upper airways—which is the case in newborns and small infants [14,19]—the higher the contribution of the above mechanism. Our simulations also revealed that a Ti of 300 ms resulted in the lowest FiO_2,_ irrespective of cannula flow (Figure 1). However, Ti values of around 300 ms represent the lower limit of normal in early infancy [16,17,18,19]. Therefore, using Benaron and Benitz’s formula with a fixed Ti of 300 ms (i.e., the STOP-ROP approach [11]) resulted in a marked underestimation of the effective FiO_2_, especially in the case of lower VTs (Figure 1). The risk of oxygen overexposure and the potential consequences for premature and most vulnerable infants are apparent.

As mentioned above, different VT, Ti, and Te combinations may result in similar MV but different FiO_2_ values. This was particularly evident in our study at lower MV and higher cannula flows (e.g., at MVs < 700 mL/min for a cannula flow of 0.1 L/min and at MVs < 1500 mL/min for a cannula flow of 1 L/min) (Figure 4). Therefore, Finer’s formula, which is based solely on MV without considering the Ti (Figure 1), would expectedly result in inaccurate FiO_2_ predictions. Indeed, Finer’s equation significantly overestimated the effective FiO_2_ in our study, and, as expected, the degree of overestimation was higher at lower MV values and higher cannula flows (Figure 6). Our findings suggest that when Finer’s formula is used to calculate effective FiO_2_ in small infants with increased oxygen demands (i.e., more severe respiratory disease), the true oxygen delivery to the lungs may be suboptimal. 

Arguably, the existing mathematical models [3,4,12] would be more accurate if the precise values of VT, Ti, and Te were available. However, such measurements are laborious and cannot be routinely performed in clinical practice [21]. Less-invasive techniques of tidal-breathing monitoring, such as impedance pneumography [22], are evolving but require specialized equipment and expertise. Thus, the only practical alternative would be to predict a range of expected FiO_2_ values (instead of a single value), ideally using simple and easy-to-obtain clinical parameters. Based on our simulations, we developed a series of predictive charts that may offer realistic FiO_2_ estimates based on cannula flow, infant body weight, and RR (Figure 7). According to these charts, the effective FiO_2_ increases as the cannula flow increases or infant weight and RR decrease (Figure 7). The latter associations are justified because infant weight (through the weight-dependency of VT) and RR are the parameters that determine the MV. Our findings are in line with a study showing that higher RRs are associated with lower hypopharyngeal FiO_2_ levels [23], while our charts accurately predict the variable hypopharyngeal FiO_2_ values reported previously by others [6]. 

This study has limitations. First, the simulations were performed by keeping the respiratory parameters constant and, thus, we could not account for the inherent variability of breathing [18]. Nevertheless, when many breaths are considered, VT, Ti, and RR vary around an average value, which may be regarded as representative of tidal breathing dynamics [21]. Second, our experiments did not account for gas exchange and oxygen consumption in the lungs. Thus, the gas exhaled from the respiratory compartment contained more oxygen than expected in vivo, which likely biased the measured FiO_2_ towards higher values. However, the effect of the above mechanism has been assessed in a previous bench study and was found to be small (i.e., FiO_2_ bias 0.7–1.6%) [12]. Finally, in our simulations, the oxygen concentration of the supplied gas was 100%. Therefore, our FiO_2_ charts are not applicable when using oxygen blending systems. Oxygen blenders provide a controlled mixture of oxygen diluted with air, allowing precise titration of the amount of delivered oxygen [24]. However, these devices are more demanding (e.g., require compressed medical air) and expensive and, thus, remain generally inaccessible to resource-limited settings [24,25]. Moreover, although oxygen blending systems protect against oxygen overexposure (i.e., the FiO_2_ delivered to the lungs cannot exceed the FiO_2_ set on the blender–FiO_2 [blender]_), the effective FiO_2_ is lower than the FiO_2 [blender]_ and, thus, may be suboptimal. In such cases, our FiO_2_ charts may still provide more realistic FiO_2_ estimates by applying the following equation: effective FiO_2_ = 21 + (effective FiO_2 [chart]_ − 21) × FiO_2 [blender]_/100, where effective FiO_2 [chart]_ is the effective FiO_2_ predicted by our simulations (Figure 7). For example, if the effective FiO_2 [chart]_ at a given cannula flow is 30%, for a FiO_2 [blender]_ of 50%, the effective FiO_2_ would be 21 + (30 − 21) × 50/100 or 25.5%. 

## 5. Conclusions

In this study, through realistic mechanical simulations, we showed that the FiO_2_ delivered by LFNC to small infants critically depends on respiratory parameters such as VT, Ti, and RR. However, since the exact VT and Ti values are not available in clinical practice, the existing mathematical formulas cannot reliably estimate the effective FiO_2_. Particularly at a lower MV, the FiO_2_ delivered to the lungs may be significantly either underestimated (Benaron and Benitz’s formula) or overestimated (Finer’s formula), thus putting small and most vulnerable infants at an increased risk of complications related to oxygen overexposure or suboptimal oxygen supply, respectively. Based on our simulations, we developed predictive FiO_2_ charts that could assist healthcare professionals in optimizing oxygen delivery by LFNC using simple and easy-to-obtain clinical parameters, such as infant body weight and RR. 

## Figures and Tables

**Table 1:** Range of simulated values.

Parameter	Simulated Range
Nasal cannula flow (L/min)	0.1–1
VT (mL)	5–80
RR (breaths/min)	30–80
Ti (ms)	300–900
Ti/Te ratio	0.5–1.5
MV (mL/min)	150–6400

## Data Availability

A complete description of the mechanical breathing simulator is openly available in the GitHub repository: https://github.com/arisberd/Infant-breath-mechanical-simulator (accessed on 2 March 2024). Due to privacy reasons, the raw data supporting this article’s conclusions are available upon request from the corresponding author.
